# Alteration of Tissue Marking Dyes Depends on Used Chromogen during Immunohistochemistry

**DOI:** 10.3390/cells10040835

**Published:** 2021-04-08

**Authors:** Selina Kiefer, Julia Huber, Hannah Füllgraf, Kristin Sörensen, Agnes Csanadi, Maren Nicole Stillger, Martin Werner, Hans-Eckart Schaefer, Peter Bronsert, Konrad Aumann

**Affiliations:** 1 Institute for Surgical Pathology, Medical Center-University of Freiburg, Breisacherstr. 115A, 79106 Freiburg im Breisgau, Germany; Julia.Huber@uniklinik-freiburg.de (J.H.); Hannah.Fuellgraf@uniklinik-freiburg.de (H.F.); kristin.werner@uniklinik-freiburg.de (K.S.); Agnes.Csanadi@uniklinik-freiburg.de (A.C.); maren.stillger@mol-med.uni-freiburg.de (M.N.S.); martin.werner@uniklinik-freiburg.de (M.W.); Hans-Eckart.Schaefer@uniklinik-freiburg.de (H.-E.S.); peter.bronsert@uniklinik-freiburg.de (P.B.); konrad_aumann@web.de (K.A.); 2 Comprehensive Cancer Center Freiburg, Medical Center-University of Freiburg, 79106 Freiburg im Breisgau, Germany; 3 Faculty of Biology, University of Freiburg, 79106 Freiburg im Breisgau, Germany; 4 Institute of Molecular Medicine and Cell Research, Medical Center, University of Freiburg, 79106 Freiburg im Breisgau, Germany; 5 Center for Pathology Kempten Allgäu-Klinikverbund Allgäu, 87439 Kempten, Germany

**Keywords:** immunohistochemistry, tissue marking, surgical margins, margin inking, gross section, DAB, Diaminobenzidine-tetrahydrochloride-dihydrate

## Abstract

Pathological biopsy protocols require tissue marking dye (TMD) for orientation. In some cases (e.g., close margin), additional immunohistochemical analyses can be necessary. Therefore, the correlation between the applied TMD during macroscopy and the examined TMD during microscopy is crucial for the correct orientation, the residual tumour status and the subsequent therapeutic regime. In this context, our group observed colour changes during routine immunohistochemistry. Tissue specimens were marked with various TMD and processed by two different methods. TMD (blue, red, black, yellow and green) obtained from three different providers (A, B and C, and Whiteout/Tipp-Ex^®^) were used. Immunohistochemistry was performed manually via stepwise omission of reagents to identify the colour changing mechanism. Blue colour from provider A changed during immunohistochemistry into black, when 3,3′-Diaminobenzidine-tetrahydrochloride-dihydrate (DAB) and H_2_O_2_ was applied as an immunoperoxidase-based terminal colour signal. No other applied reagents, nor tissue texture or processing showed any influence on the colour. The remaining colours from provider A and the other colours did not show any changes during immunohistochemistry. Our results demonstrate an interesting and important pitfall in routine immunohistochemistry-based diagnostics that pathologists should be aware of. Furthermore, the chemical rationale behind the observed misleading colour change is discussed.

## 1. Introduction

A distinct determination of surgical resection margin is essential for pathological diagnoses, particularly regarding oncological specimens [1,2,3,4]. For instance, in breast cancer, resection margin status and use of adjuvant chemotherapy are the most important factors associated with local recurrence and patient survival [5]. Use of tissue marking dye (TMD) enables a correct spatial orientation of complex surgical specimens and a reliable assessment of surgical resection margins microscopically. Thus, detailed reporting of tumour distance from the resection margins is possible. Therefore, use of TMD is crucial for a complete pathological report including exact information regarding surgical excision status which have major impact on the recurrence rate [6,7,8,9].

Several marking techniques are proposed in the literature [10,11,12]. The most commonly used colour is “India ink” [13,14]. As alternative methods, alcian blue solution or Whiteout (Tipp-Ex^®^), which was mentioned in 1990 for example by Harris [15], are described. Another option to mark resection margins is starch, though this method has already been discussed as unreliable on ragged or partly disrupted tissue in 1991 by Hunter-Craig C. et al. [11]. However, these methods generate a single colour only [13], which is not sufficient in the case of more complex resection specimens (e.g., breast cancer lumpectomy).

The requirements on TMD are broad. The staining substance has to be microscopically bright and should not smudge into surrounding tissue or mix with other applied colours. The TMD should be easily applicable and stick to both fresh and formalin fixed tissue specimens. Different conditions of the tissue (e.g., fatty or parenchymatous) should not influence the properties of the colour. Additionally, TMD should be cost effective and commercially available. The most important property of TMD should be their colour fastness. No colour change during routine histopathology workup, as no fade or vanishing of colour should occur as this could lead to severe misinterpretation of resection margins with fatal consequences.

The aim of the current study was to examine the fastness and fidelity of commercially available TMD during routine tissue processing and immunohistochemical analyses. Therefore, TMD from three different providers (see Section 2) and Whiteout have been tested and evaluated according to a specified protocol.

## 2. Materials and Methods

### 2.1. Tissue Specimen

Formalin fixed (4% buffered formaldehyde) and paraffin embedded surgical specimens of different tissues (breast, kidney and tissue specimens with active endometrioses comprising haemorrhages) that were not required for further diagnostic steps were used. All tissue specimens were cut to a similar size and triangle shape.

### 2.2. Tissue Marking

TMD from The Davidson Marking System^®^ (Bradley Products Inc., Bloomington, MN, USA (A)), Thermo Scientific^®^ (Tissue Marking Dye TMD, Kalamazoo, MI, USA (B)) and Cancer Diagnostics Inc (CDI’s Tissue Marking^®^, Morrisville, NC, USA (C)) were used (product numbers available upon request).

**Figure 1:**
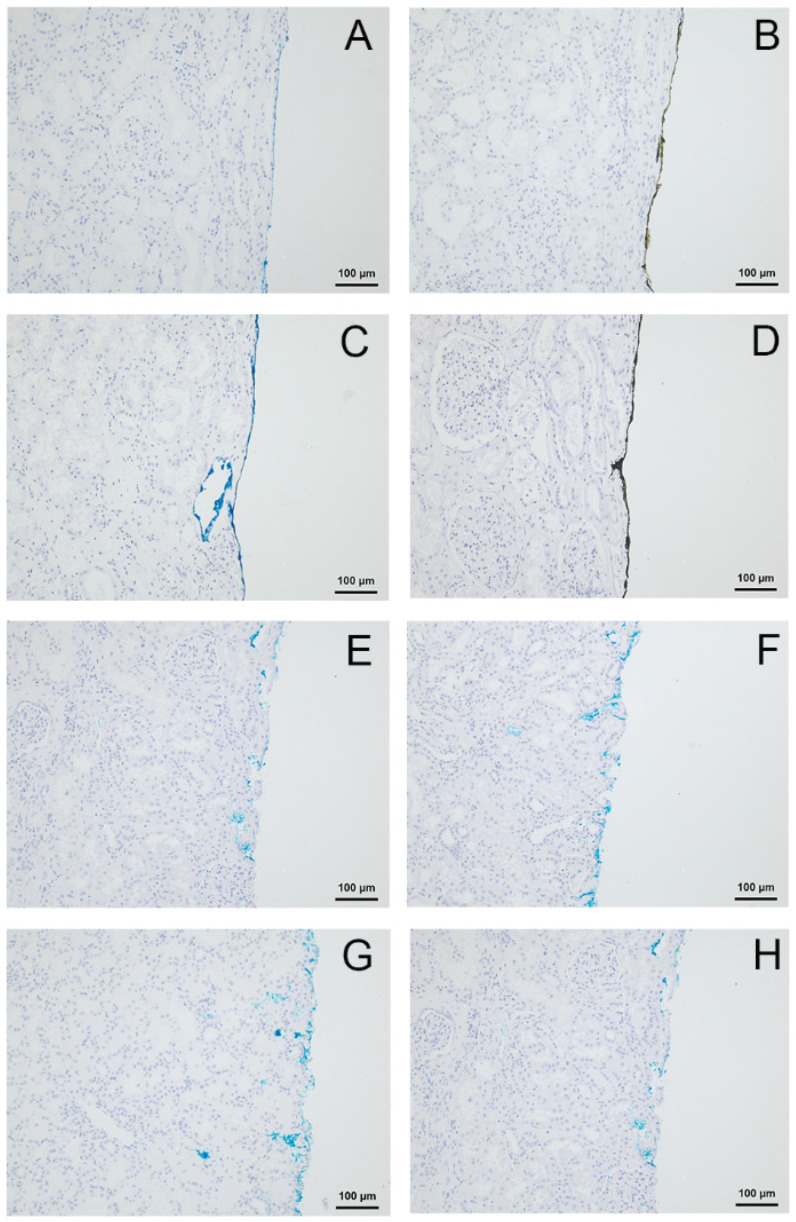
Colour change of tissue marking dye (TMD) from provider A during immunohistochemistry was not observed with Dako K5005 (**A**) but with Dako K8002 containing 3,3′-Diaminobenzidine-tetrahydrochloride-dihydrate (DAB) (**B**). No colour change of TMD from provider A by treatment with Kit A (**C**) but by treatment with Kit B (**D**) containing DAB. The marking dyes from provider B (**E**,**F**) and provider C (**G**,**H**) remained unchanged with Dako K5005 (**E**,**G**) as well as with Dako K8002 (**F**,**H**).

For each provider, a set of four specimens of breast and four of kidney tissue was prepared. Subsequently, each set was split into two subsets for tissue processing. Each subset was marked with the colours blue, black, green, red and yellow from the providers and with Whiteout (Tipp-Ex^®^, Societe BIC, S.A., Clichy Cédex, France) (Figure 1).

### 2.3. Tissue Processing

After preparation of the tissue specimens, one subset was processed using Shandon Excelsior TM ES (xylene-based system) (I) and the other (corresponding) subset was processed using Leica PELORIS II (vacuum/ethanol-based system) (II).

### 2.4. Histochemistry and Immunohistochemistry

All samples were cut into 2 µm thick slices. Prussian blue staining was performed according to standard protocols [16]. Immunohistochemistry was performed with two standard immunohistochemical antibodies-Cytokeratin 5/6 (CK 5/6; DAKO IR780) and Cytokeratin 7 (CK7; DAKO IR619). All staining was performed with Dako AutostainerPlus. The detection systems Dako Envision Flex+, Mouse, High pH (Link) Detecting System (K8002; containing < 10% 3,3′-Diaminobenzidine-tetrahydrochloride-dihydrate (DAB)) and Dako real Detecting System Alkaline Phosphatase/RED (Dako K5005) including Link biotinylated secondary Antibody Streptavidin Alkaline Phosphatase, were used.

To test the influence of the chemical reagents of the Dako Envision Flex-kit on the TMD properties, stepwise omission of the required reagents was conducted. Additionally, all reagents were applied individually.

### 2.5. Enzyme Cytochemistry

To check a potential cross-reaction between reagents used for antibody detection and the TMD, colour marked breast and kidney tissues were treated with either hydrogen peroxide (H_2_O_2_) and horseradish peroxidase (HRP) (Mix A), H_2_O_2_ and Diaminobenzidine-tetrahydrochloride-dihydrate (DAB; Mix B) or DAB and HRP (Mix C); each of them in the concentrations as provided by the supplier (Dako).

The influence of a peroxidase-like reaction (pseudo-peroxidase) on the staining properties of the TMD was checked by performing a Prussian blue staining (Perls’ reaction) [16]. To this end, tissue specimens with active endometriosis comprising haemorrhages with hemosiderin deposits were first reacted with acidified potassium ferrocyanide (Perls reagent) to obtain a Prussian blue staining. Subsequently, DAB-based peroxidase reaction was applied to Prussian blue marked tissue samples.

Additionally, the blue TMD from providers A, B and C were processed, as mentioned above, without tissue specimen. To this end, dots of the TMD were spotted onto glass slides. These slides were treated either with DAB only, Mix A, Mix B or Mix C.

## 3. Results

Each of five different TMD (blue, black, green, red and yellow) of three different providers (A, B, C) were investigated in samples rich in adipose tissue (breast) and parenchymatous tissue (kidney) regarding their colour fastness during immunohistochemistry. The slides were assessed independently by two pathologists (SK and HF), blinded to the type of tissue, the performed protocols, the TMD provider and the applied TMD colour. The pathologists noted the perceived colours on each slide. After completed reviews, their perceived colours were compared to the applied TMD.

### 3.1. Colour-Changes

During immunohistochemistry, the TMD kept their properties using the detection system Dako K5005. No difference of the applied colour and the colour perceived was observed. However, using the detection system Dako K8002, a change of the colour blue into black was observed by both pathologists independently. This was true only for the TMD blue from provider A. No colour change of the colour blue from providers B and C (Figure 1) was observed.

No colour changes were observed for the other TMD (green, red, yellow or black) from provider A. The different tissue processing (I and II) had no effect on the colour. By stepwise omission of reagents of the immunohistochemical procedures, colour changing from blue to black was only observed in the presence of DAB and H_2_O_2_. Strikingly, omitting DAB only led to the absence of the observed colour change.

An overview about the present or absent of the colour change of the colour blue from the different providers (A, B, C) depending on the immunohistochemical processing, is shown in Figure 1.

To figure out whether DAB causes a particular reaction with the blue TMD from provider A or whether it is a normal oxidation reaction, two other reagents with oxidation potential were tested (H_2_O_2_ and HRP, in Mixes A, B and C as described above). The colour-marked histological slides (kidney parenchyma) showed a change of blue (provider A) into black colour when treated with Mixes B or C. Both mixes contained DAB. The treatment with Mix A, without DAB, did not cause any colour change (Figure 1).

To check if the colour change is tissue-dependent, drops of the three different blue TMDs without tissue were applied on glass slides. These were treated with the three different mixes (Mix A, Mix B and Mix C). As a result, slides with a drop of the blue TMD from provider A demonstrated a colour change to a green-brown colour if the mix contained DAB (Mixes B and C). The blue TMD of providers B and C did not demonstrate any colour changes. This effect was independent of the presence or absence of DAB.

Whiteout (Tipp-Ex^®^) displayed a black colour after immunohistochemical processing regardless of the composition of the detection reagents.

**Figure 2:**
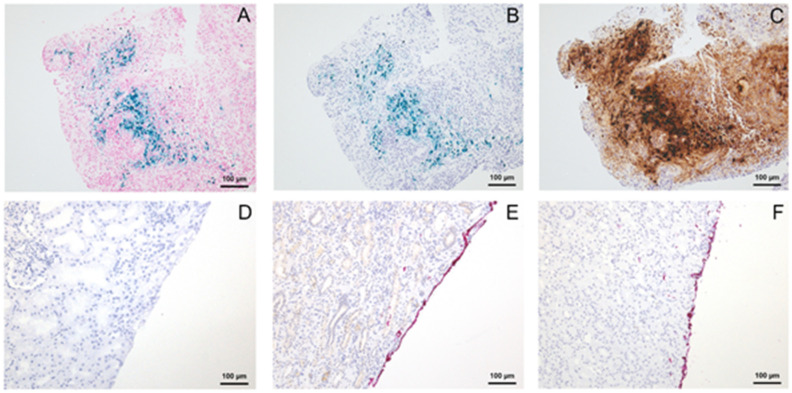
Tissue specimens of haemorrhagic endometriosis using the Perls’ reaction (**A**), Kit A (**B**) or Kit B (**C**). Vanishing of red colour from provider A (**D**) and intensity of red colour from providers B (**E**) and C (**F**) after immunohistochemistry in kidney tissue.

For the observed DAB-dependent colour change of the blue TMD of provider A, we suspected iron to be the potential trigger. Therefore, we stained tissue specimens of endometriosis with haemorrhages using the Perls’ reaction. As expected, siderin iron reacted blue. By adding DAB alone or Mixes B and C (which also contains DAB), the blue stained iron also turned into black, whereas applying Mix A (which contains no DAB) did not cause any change of the Prussian blue positive pigment (Figure 2).

### 3.2. Colour Intensity

Additionally, the colour intensity of the used TMDs has been evaluated independently by two pathologists (SK, HF) by scoring the intensity of the applied TMD semi-quantitatively (weak, moderate, strong). Surprisingly, after immunohistochemical processing, the red TMD from provider A could not be seen anymore by both pathologists. It vanished completely or nearly completely, regardless of the tissue type (fat cell rich or parenchymal specimens) and the presence of DAB. In contrast, the intensity of the red TMD from providers B and C did not fade and the TMD remained visible (Figure 2).

## 4. Discussion

The aim of this study was to examine the fastness and fidelity of commercially available TMD during routine tissue processing and immunohistochemical analyses. There are a few studies in the literature, which already analysed the properties of TMD [2,3,4,5,14]. All of these studies investigated TMD in H&E stained histological slides. There is only one study in the literature which analysed TMD in the context of immunohistochemical processing [17].

In our study, we observed a DAB-dependent colour change of the blue TMD from provider A after immunohistochemical staining procedure. None of the other tested TMDs from providers A, B and C showed any colour change. Similar to our results, the study of Williams et al. in 2014 [17] reported a colour change from blue to black after immunohistochemistry by using the blue TMD of provider A. The authors mentioned a “chemical reaction” that causes the colour change from blue to black. However, deeper analyses of this “chemical reaction” are still missing. To understand the colour change, we compared fat cell rich (breast) and parenchymal (kidney) tissue specimens, as well as different tissue processing protocols, to identify the crucial step in immunohistochemical treatment provoking colour change. In addition, we aimed to specify the “chemical reaction” [17] that causes the colour change from blue to black. To this end, we performed a step-by-step immunohistochemical treatment that identified Diaminobenzidine-tetrahydrochloride-dihydrate (DAB) as the responsible reagent. Both, the complete immunohistochemical staining procedure as well as the staining procedures omitting one of the reagents except for DAB caused the observed colour change. Except DAB, no other reagent applied for immunohistochemical staining caused a change of colour, regardless of tissue processing method and tissue characteristics (fatty/parenchymatous). The hypothesis that DAB + H_2_O_2_ is the responsible reagent for the colour change is supported by our observation that no colour change occurred when using the detection kit K5005 from Dako. The K5005 system uses fast red salt in an alkaline phosphatase reaction instead of DAB as chromogen and H_2_O_2_ in peroxidase-based detection system. To confirm that DAB triggers the observed colour change, we performed tests where we incubated different TMDs with DAB alone and together with H_2_O_2_ or HRP, resulting in the same colour change.

In 1970, Schaefer and Fischer [18] demonstrated a similar effect for Sudan Black staining. By adding HRP to Sudan Black with H_2_O_2_, a colour change from blue-black into a brown-black colour was observed. In this reaction, Sudan Black acts as an electron donor and becomes oxidized into a dark brown polymeric compound insoluble in alcohol or xylene as the basis for the so called stable sudanophilia, a term coined by Lillie and Burtner [19] indicating that several lipid dyes change into insoluble compounds on oxidation. As one of the reasons for the colour change, the authors mentioned that the main component of the stain is composed of an aromatic amine. In our case, adding HRP and H_2_O_2_ only caused a colour change when adding DAB. DAB includes an aromatic diamine, which might be responsible for the colour change in TMD based on Prussian blue as a signal colour. Our hypothesis that Prussian blue exerts a pseudo-peroxidase reaction is supported by the tests we performed with endometriosis tissue, where we also observed a colour change by adding DAB + H_2_O_2_. Due to this, a cross-reaction of DAB with Prussian blue (Fe_4_[Fe(CN)_6_]_3_) or a similar iron compound as a plausible ingredient of the blue TMD from provider A appears reasonable. The use of Prussian blue suspended in gelatine as a marking system has a more than 100 years lasting history; von Kupffer [20] used this paint to visualize gall capillaries. A pseudo-peroxidase activity of Prussian blue is a well-established fact, which has been exploited, for instance, to trace minimal amounts of Fe^3+^ in tissue samples [21,22].

Unfortunately, we could not get further information about the ingredients of the blue TMD from provider A. So far, it was not possible to verify our hypothesis of cross-reaction of DAB with an iron compound of the TMD.

In our study, the red TMD from provider A vanished completely or almost completely after immunohistochemical processing. The same effect was observed by Kosemehmetoglu et al. [13], although with another red TMD, from a different provider. To improve the adherence between the TMD and the tissue specimen, several authors have suggested different approaches [3,14].

Parkinson et al. suggested a drying phase of approximately 10 min. After this drying phase, the TMD would not be so easily washed off during routine processing [23]. With regard to the constantly increasing amount of tissue specimen and time pressure, which is a clear issue for every modern pathological institution, extending the drying phase would not be an option in daily routine diagnostics. Moreover, a drying phase of 10 min would surely have a negative effect on the condition of the tissue and decreases the quality of the histological results.

Whiteout (Tipp-Ex^®^) turned out to be a suboptimal alternative for marking resection margins when using several colours. It turned from white to black during histological or immunohistochemical processing which could lead to a misinterpretation of the resection margin or confusion when additional black TMD is used. If only one colour is necessary, it might be an option.

In pathology diagnostics, besides immunohistochemistry, also fluorescence in situ hybridization (FISH) assays are in use. One study describes auto-fluorescence of several TMDs during FISH [24]. Although not included in our study, we expect no colour change during FISH, due to the missing of DAB as a reagent. 

Moreover, when using new technologies, the colour fastness of TMD is important as well. A study of Hon et al. [25] reported a misinterpretation of TMD by using digital pathology techniques.

**Figure 3:**
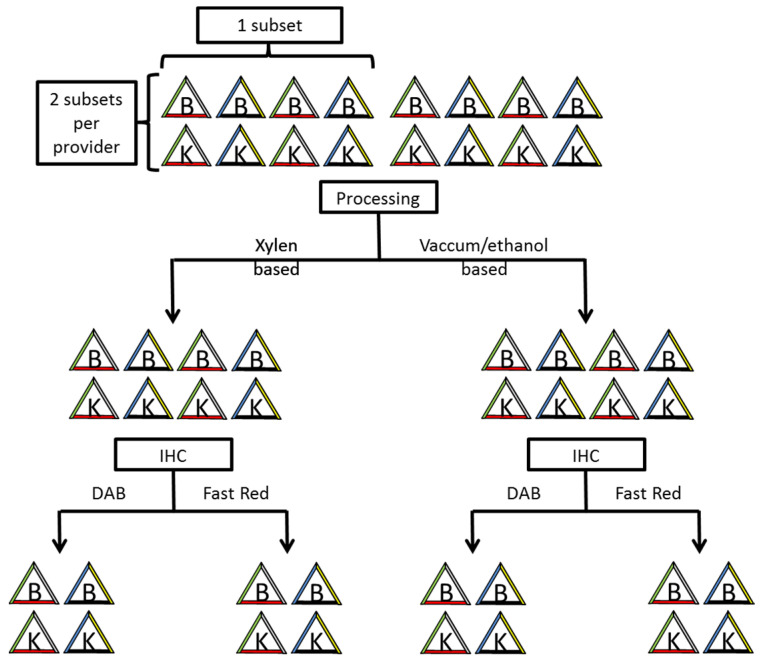
Suggested workflow for evaluation of TMD colour fastness.

As a conclusion of our study for pathological diagnostics, we note that it is important to be aware of the possible colour change or total vanishing of TMD of colour-marked surgical margins during immunohistochemical procedure. Whenever using commercially available TMD, each laboratory should evaluate the products on colour fastness during immunohistochemical processing. We suggest the workflow described above and visualized in Figure 3 to evaluate the colour fastness of the TMD. To avoid misinterpretation when assessing colour-marked surgical margins of tissue probes after immunohistochemical staining, a comparison with H&E stained slides is also recommended. Furthermore, our data help pathologists to figure out which TMD is compatible with their IHC detection kits and reagents.

Two subsets of triangular-shaped tissues marked with a different colour on each side. Two different processing (Xylen and Vaccum/ethanol based) and IHCs with two different chromogens (DAB and Fast Red) per provider. 

## Data Availability

The data presented in this study are available on request from the corresponding author (SK). The data are not publicly available due to containing information that could compromise the privacy of research partivipants.

## Abbreviations

K	Kidney
B	Breast
IHC	Immunohistochemical treatment
DAB	3,3′-Diaminobenzidine-Tetrahydrochloride-Dihydrate
